# Composite Polymer Granules Based on Poly-ε-Caprolactone and Montmorillonite Prepared by Solution-Casting and Melt Extrusion

**DOI:** 10.3390/polym15204099

**Published:** 2023-10-16

**Authors:** Anna Sukhanova, Anatoly Boyandin, Natalya Ertiletskaya, Taisia Shalygina, Alexander Shabanov, Alexander Vasiliev, Ivan Obvertkin, Valeria Brott, Yulia Prokopchuk, Alexander Samoilo

**Affiliations:** 1 Department of Biodegradable Polymers Materials, Reshetnev Siberian State University of Science and Technology, 31 Krasnoyarskiy Rabochiy Av., Krasnoyarsk 660037, Russia; boyandin@biopolymer.pro (A.B.); natalya.ertiletskaya@gmail.com (N.E.); valerie_brt@mail.ru (V.B.); batori_bloody@mail.ru (Y.P.); x_lab@rambler.ru (A.S.); 2 Department of Analysis, Synthesis, Modeling and Digital Design of Smart Materials with Specified Properties, Reshetnev Siberian State University of Science and Technology, 31 Krasnoyarskiy Rabochiy Av., Krasnoyarsk 660037, Russia; leonova.ta@inbox.ru; 3 Department of Molecular Spectroscopy, Kirensky Institute of Physics, Federal Research Center “Krasnoyarsk Science Center SB RAS”, 50/38 Akademgorodok, Krasnoyarsk 660036, Russia; alexch_syb@mail.ru; 4 Department Engineering Physics and Radio Electronics, Siberian Federal University, 79 Svobodny Av., Krasnoyarsk 660041, Russia; adva@iph.krasn.ru; 5 Department of Digital Design of Transformable Structures based on Smart Materials, Reshetnev Siberian State University of Science and Technology, 31 Krasnoyarskiy Rabochiy Av., Krasnoyarsk 660037, Russia; 79632609742@ya.ru; 6 Department of Scientific Activities, Science and Technology, Siberian Federal University, 79 Svobodny Av., Krasnoyarsk 660041, Russia; 7 Department of Intelligent Materials and Systems, Reshetnev Siberian State University of Science and Technology, 31 Krasnoyarskiy Rabochiy Av., Krasnoyarsk 660037, Russia

**Keywords:** biodegradable polymers, poly-ε-caprolactone, montmorillonite, polymer composites, processing technologies

## Abstract

Eco-friendly polymer composites in the form of granules based on biodegradable polycaprolactone (PCL) with the inclusion of montmorillonite (MMT) from 5 to 50 wt% were prepared by solution-casting and melt extrusion. The physicochemical properties of the composite granules were studied using FTIR spectroscopy, XRDA, DSC, and TGA methods. The paper presents comparative values of crystallinity of composite granules which depend on the method of measuring (XRDA, DSC). It was shown that the crystallinity of PCL/MMT granules was affected by the preparation method and by the MMT content, and that with increase in MMT content, crystallinity increased by up to 61–67%. The change in crystallinity of the granules also affected its biodegradation in soil. At the end of exposure in soil, the mass loss for the granules prepared by solution-casting was more than 90%, whereas for the composite granules prepared by extrusion it was less than 60%. Applying melt extrusion enabled obtaining intercalated composites with predictable features, whereas only mixed-structure microcomposites could be prepared by solution-casting.

## 1. Introduction

Recently, biodegradable polymers have attracted considerable interest in the packaging industry and in agriculture as they can be used as a means of reducing environmental pollution from plastic waste [1,2,3]. However, for the wide usage of biodegradable polymers, they must have low costs and exhibit reasonable mechanical properties compared to conventional non-biodegradable materials.

Poly-ε-caprolactone (PCL) is a biodegradable polyester synthesized from an ε-caprolactone monomer. This polymer is considered to be an excellent candidate for application in the packaging industry due to its physical properties and commercial availability. The degradability of PCL allows it to be used for the development of eco-friendly packaging and mulches, as well as carriers for pesticides and fertilizers in agriculture. For example, Yang et al. (2022) developed biodegradable mulching films based on PCL. The resulting PCL films with a nanoporous structure showed improved temperature properties and good wettability and were proposed to be used for conserving soil moisture and suppressing weeds. It is worth noting that the authors used a non-standard method for the producing of films by thermally induced phase separation (TIPS) to fabricate PCL films in order to attain their porosity [4]. Also of interest is a recent study on the production of melt-processed PCL/hydroxyapatite films loaded with urea, which, the authors claim, can be used in agricultural applications for minimizing the plastic pollution generated from the agricultural sector, and benefitting the soil through the leaching of both urea and hydroxyapatite during the assimilation of the PCL matrix [5].

However, the use of PCL is limited by its poor processability and slow crystallization rate. To improve the performance of PCL, it is often blended with other polymers, such as poly(3-hydroxybutyrate) [6], polylactic acid [7], starch [8], cellulose [9] or inorganic fillers [10]. Montmorillonite, bentonite, saponite, hectorite, smectite and laponite are most commonly used as clay mineral fillers [11,12,13,14,15]. One of the most widely used types of PCL filler is montmorillonite (MMT), which is a layered silicate in which ions can be replaced by organo-ions in order to increase the distance between the layers and to improve polymer/clay compatibility [16]. When the clay is introduced into the polymer matrix, materials of three different groups can be potentially obtained. These are phase-separated, intercalated and exfoliated composites [17]. If macromolecules do not penetrate the interlayer space of the clay, then the clay is presented in the polymer matrix in the form of a packed structure called a tactoid, and a phase-separated microcomposite is formed as a result. If the macromolecules or their fragments are positioned in the gaps between the individual layers of clay, intercalated nanocomposites are formed as a result of compounding. An exfoliated nanocomposite is formed when the clay layers are separated by polymer interlayers that are evenly distributed in the polymer matrix [18]. In normal conditions such composites notably feature mixed morphology, which is mostly affected by the nature of the clay and the method of obtaining the composites. The methods of obtaining the polymer/clay composites are largely divided into three main groups: introduction of the filler into the polymer solution, polymerization (in situ polycondensation), and blending in the polymer melt. As shown in [19,20,21], it is possible to obtain microcomposite systems by blending melted PCL with unmodified MMT, whereas intercalated systems can be obtained by blending with melted MMT modified with various alkylammonium cations. Moreover, the ε-caprolactone monomer can be blended with MMT and polymerized at room temperature in the presence of dibutyltin dimethoxide as a polymerization catalyst [22], or by including a second more polar organomodifier with a much shorter chain length [11].

The inclusion of micro- or nanoparticles of clay was shown to have variable effects on the degree of crystallinity, size, shape and morphology of crystals and/or the kinetics of crystallization of the pure matrix [15]. This phenomenon was described by Di Maio et al., who claimed that clay particles can both increase and decrease the crystallization rate of semi-crystalline PCL [23]. A similar effect was observed in the study by Barabaszova et al. According to FTIR analysis of films based on PCL and vermiculite prepared by solution-casting, the height of the PCL absorption bands decreased depending on the concentration of vermiculite and its treatment [12]. In a study by Luduena et al., the type of clay (modified or unmodified) and its distribution in the polymer matrix also affected the crystallinity of the melted PCL granules. When MMT was added, the crystallinity of PCL dropped to 49%, but when Cloisite 30B (organomodified MMT) was added, the crystallinity increased to 53% compared to the pure PCL matrix [24]. In a comprehensive study of the rheological properties of PCL and PCL-clay blends, it was found that the inclusion of clay over a short time resulted in a significant improvement in the creep resistance, which also affected the crystallization rate of the PCL [25]. Another study revealed that PCL itself acted as a compatibilizer for the production of polymer-clay composites based on ABS, PP and PE [26].

It is worth noting that, despite a great quantity of published data on the processing, structure and characteristics of PCL/clay composites obtained by different techniques, data on the effect of the preparation method on crystallization and the characteristics of PCL and its composites are very limited and mainly address only certain types of polymer composites. Comparison of the influence of different production methods is important because it enables understanding of how a particular method of producing a composite affects its properties and, accordingly, enables choice of the appropriate one depending on the application of the final product. This paper presents a comparative study of the effect of the filler concentration and preparation method (solution-casting and melt extrusion) on the structure, crystallinity and thermal characteristics of polymer composites based on PCL with different MMT content.

## 2. Materials and Methods

### 2.1. Materials and Obtaining of Composites

The studied biodegradable polymer poly-ε-caprolactone (PCL) in the form of granules had a weight-averaged molecular weight (*M_w_*) value of 80 kDa, a degree of crystallinity (*C_x_*) value of 52% and a melting point (*T_m_*) value of 90 °C, and was purchased from Sigma Aldrich (St. Louis, MO, USA). The molecular weight was determined using an Agilent Technologies 1260 Infinity high-performance liquid chromatographer (Waldbronn, Germany) supplemented with Agilent PS-H EasiVial calibration standards. Nanosilicate–organophilic clay Monamet-101 (Metaclei, Karachev, Russia) with a particle size of <165 μm, which constituted the chemically modified montmorillonite (MMT), was used as a filler material. Reagent grade chloroform (Ecos-1, Sochi, Russia) was used as a polymer solvent.

To obtain composite granules by solution-casting, MMT was first introduced into a 3% solution of PCL in chloroform in the ratios of 5, 10, 25 and 50 wt% of the polymer. The resulting mixture was stirred on a magnetic stirrer for 24 h at 3000 rpm. The stirred mixture was sucked into a syringe, followed by ejecting onto the surface to form a filament, and left to dry in a fume hood. The resulting filament was cut into granules manually.

Melt-extruded PCL\MMT granules were obtained using a Brabender 19/25D single-screw extruder (Brabender GmbH, Duisburg, Germany) with three heating zones. The polymer and clay were preliminarily blended in a Brabender 50 EHT (Brabender GmbH, Germany) measuring mixer in the same ratios as for the solution-casting. The temperature profile used was 80–90–100 °C. The resulting filament was then granulated using a Brabender laboratory pelletizer, type 12-72-000 (Brabender GmbH, Germany).

For further analysis, the obtained samples were given the following designations: S_PCL/MMT 5%, S_PCL/MMT 10%, S_PCL/MMT 25%, S_PCL/MMT 50%—for granules prepared by solution-casting; E_PCL/MMT 5%, E_PCL/MMT 10%, E_PCL/MMT 25%, E_PCL/MMT 50%—for granules prepared by melt extrusion. The original PCL granules (neat PCL) were used as control samples.

### 2.2. SEM (Scanning Electron Microscopy)

The microstructure of cross-sections of the composites and of Monamet-101 were studied using scanning electron microscopy (TM-4500 microscope, equipment of the Krasnoyarsk Regional Center of Research Equipment of the Federal Research Center “Krasnoyarsk Science Center SB RAS”, Hitachi, Tokyo, Japan). Prior to this, the samples were coated with platinum (at 10 mA, for 45 s) with an Emitech K575X sputter coater (Leica, Vienna, Austria). The micrographs were taken at ×100 magnitude.

### 2.3. FTIR (Fourier Transformed Infra-Red) Spectroscopy

The chemical structure of the samples was studied by infrared spectroscopy with Fourier transform using a Nicolet iS10 FTIR spectrometer (Thermo Scientific, Waltham, MA, USA) and an ITX Smart prefix (Thermo Scientific, USA) with a diamond crystal using a disturbed total internal reflection method (DTIR). The analyses were carried out with a spectral resolution of 4 cm^−1^ averaged over 32 scans, in the range of 4000–400 cm^−1^. To determine the crystalline to amorphous phase ratio, the PCL carbonyl band (1600–1820 cm^−1^) was analyzed, and its complex contour was deconvolved into its components [27,28,29]. The OMNIC v. 9.8.372 software was used for processing of the obtained IR–Fourier spectra and for separation of the complex contour band in the several individual band components, applying advanced correction of disturbed total internal reflection. Deconvolution of the peaks of the C=O groups allowed us to determine their area and to analyze changes to them. The degree of crystallinity *X_c_* was calculated according to the formula:

(1) $$X_{c}=\frac{A_{c}}{A_{c}+A_{a}}$$

where *A_a_* is the area of the ν(C=O)free1 peak, and *A_c_* is the area of the ν(C=O)int2 peak [29].

### 2.4. XRD (X-ray Diffraction) Analysis

To obtain X-ray spectra of the composite granules and the Monamet-101, the analyzed sample was first pressed into plates and fixed in a cuvette. Registration of the X-ray spectra was performed on a D8 ADVANCE powder X-ray diffractometer (Bruker AXS, Karlsruhe, Germany, equipment of the Krasnoyarsk Regional Center of Research Equipment of the Federal Research Center “Krasnoyarsk Science Center SB RAS”) in the area of 4 ÷ 60° angles 2θ. The radiation wavelength corresponded to the standard CuKα curve. The obtained data were processed using the Eva 5.2.0.3 complementary software that was provided with the diffractometer.

The obtained X-ray spectra of the samples were used for crystallinity calculation according to the software Formula (1), which has the form:

(2) $$C_{x}=1-\frac{S_{1}}{S_{2}}$$

where *S*_1_ is the area bounded by the amorphous background curve (amorphous halo), and *S*_2_ is the total area under the X-ray curve. The areas were calculated by integration with deduction of the instrumental background.

### 2.5. DSC (Differential Scanning Calorimetry), TGA (Thermogravimetric Analysis) and DTA (Differential Thermal Analysis)

The temperature-modulated differential scanning calorimetry of the samples was performed using a DSC25 differential scanning calorimeter (TA Instruments, New Castle, DE, USA) in standardized aluminum crucibles in an atmosphere of pure N_2_ at a flow rate of 70 mL/min. The sinusoidal heat flow was modulated with a period of 60 s and an amplitude of ±1 °C. Determination of the isothermal crystallization of the granules was performed in several steps:

1. Heating from room temperature to 100 °C at a 10 °C/min rate.
2. Melting at 100 °C for 10 min.
3. Cooling to 15 °C at a 5 °C/min rate.
4. Exposure at *T_c_* for 30 min to complete the crystallization.
5. Heating from 15 °C to 100 °C at a 10 °C/min rate.

The obtained thermograms were further processed using TRIOS v. 5.00.44608 software. For the subsequent calculations, the measurement results of the second heating segment were used.

TGA and DTA analyses were performed under the given conditions using an SDT Q600 thermal analyzer (TA Instruments, USA). The analyzed granules were fragmented with a sharp blade into particles of about 1 mm in size. Several dozen such particles were used for analysis.

### 2.6. Isothermal Crystallization Kinetics

The obtained data on isothermal crystallization were additionally analyzed in terms of Avrami’s theory of phase change according to the following Equation (2) [24,30,31,32]:

(3) $$(1-X_{t})=e^{-Kt^{n}}$$

where *X_t_* is the fractional crystallization at time *t*, *K* is the rate constant, and *n* is the exponent of the Avrami degree. The crystallization behavior is usually stated in terms of the rate constant *K* and the crystallization half-time *t*_1/2_, which is defined as the time consumed to reach half of the final crystallinity.

### 2.7. Biodegradation of the Composites in Soil

The biodegradation of the granules obtained was studied in the field soil. The experimental soil contained ≥300 mg/L of nitrogen (NH_4_ + NO_3_), ≥300 mg/L of phosphorus (P_2_O_2_), ≥430 mg/L of potassium (K_2_O), and trace minerals (magnesium, iron, boron, manganese, zinc, copper, molybdenum). The C:N ratio was 30:1. The soil pH in the salt solution was 6.5 and the humidity was 57%. A quantity of 100 mg of granules of each group was placed in plastic containers previously filled with 50 g of experimental soil and then covered with an additional 50 g of soil making 100 g of soil in total in each container. The containers were incubated at room temperature in a dry ventilated room. The humidity of the soil in the containers was maintained by regular watering (no less than once a week), with 50 mL of tap water per container. The experiment was conducted over 245 days (from August 2021 to April 2022) with 7 experimental points (30, 56, 91, 120, 161, 211 and 245 days). At each experimental point, the granules were gently removed from the container for further determination of the residual mass. The removed samples were gently washed with distilled water and dried at 50 °C in a drying chamber for 48 h. The dried samples were weighed, and the residual mass was determined according to the formula:

(4) $$m_{R}=\frac{m_{1}}{m_{0}}\times 100$$

where *m*_1_ is the mass of the degraded sample, and *m*_0_ is the initial mass of the degraded sample before incubation in the soil.

Groups of biodegraders were determined 210 days after the exposure of the granules in the soil using conventional microbiological methods. Soil suspensions at 10^−8^–10^−4^ dilution were used for plating on nutrient agar, starch–ammonia agar, Ashby’s agar and Sabouraud agar (for ammonifiers, prototrophs, assimilating mineral nitrogen, nitrogen fixers and fungi) in Petri dishes. The platings were incubated at 32 °C (the Sabouraud platings were incubated at 28 °C) over 7 days. After the incubation, the colonies appearing were counted and their numbers were used for calculation of the bacterial cell concentration in the soil (in CFU/(g·mL)) according to the formula:

(5) $$C=\frac{a\cdot b}{c\cdot d}$$

where *a* is the average number of microbial colonies on a Petri dish, *b* is the dilution used for plating, *c* is the suspension volume used for plating (mL), and *d* is the mass of the soil used for preparation of the suspension (g).

The results of the calculation were compared with the equivalent results for the initial soil (prior to granule incubation), which were also obtained according to the procedure described above.

### 2.8. Statistical Data Processing

The obtained data were statistically processed by standard methods using the Microsoft Excel 2010 software package for Windows 7. All measurements were carried out in triplicate. For the obtained data, the means, the mean squared errors, and the confidence intervals were calculated. All the calculations were carried out assuming a significance level of *α* = 0.05.

## 3. Results

### 3.1. SEM

**Figure 1:**
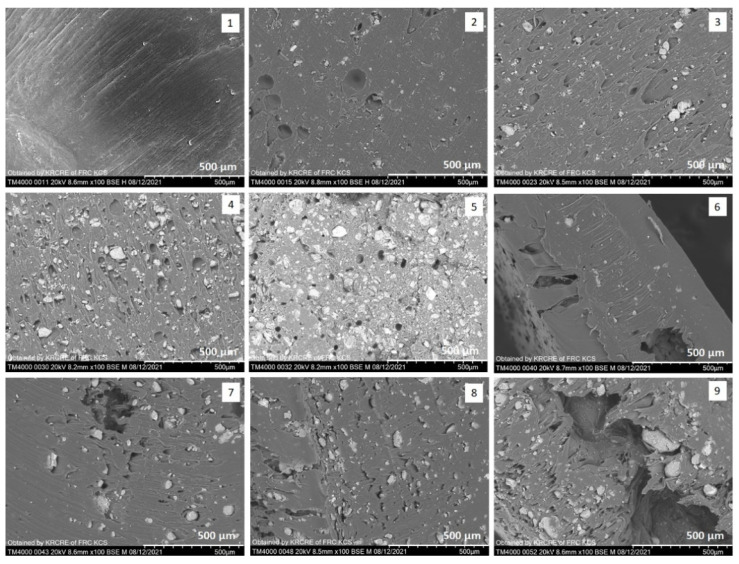
SEM images of cross-sections of PCL granules (**1**) and PCL/MMT composite granules prepared by extrusion with different MMT content: (**2**)—E_PCL/MMT 5%; (**3**)—E_PCL/MMT 10%; (**4**)—E_PCL/MMT 25%; (**5**)—E_PCL/MMT 50% and PCL/MMT composite granules prepared by solution-casting with different MMT content: (**6**)—S_PCL/MMT 5%; (**7**)—S_PCL/MMT 10%; (**8**)—S_PCL/MMT 25%; (**9**)—S_PCL/MMT 50%.

A series of PCL granules with different MMT content were obtained using solution-casting and melt extrusion. The distribution of MMT in the polymer matrix can be observed on the cross-sections of the polymer granules in Figure 1. While the cross-sections of neat PCL granules featured a smooth microstructure, the composite PCL/MMT granules were observed to have pores, craters and agglomerates of MMT. In granules prepared by extrusion, the size of the MMT particles ranged from 5 to 165 μm based on the MMT content. The E_PCL/MMT 5% granules were predominantly dominated (up to 80%) by MMT particles ranging in size from 10 to 50 μm. In the E_PCL/MMT 10% and the E_PCL/MMT 25% granules, the MMT particles were distributed in size as follows: <10 μm—11%, 11–50 μm—65%, 51–100 μm—15%, >100 μm—9%. The highest number of particles (up to 80%) with a size of 50–100 μm was observed for the E_PCL/MMT 50% granules due to the increase in the MMT content.

**Figure 2:**
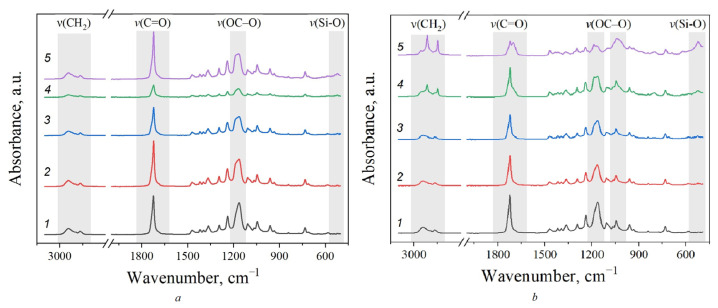
FTIR-spectra for PCL (1) and for PCL/MMT composites (2–5), prepared by solution-casting (**a**) and by melt extrusion (**b**): 1—PCL, 2—PCL/MMT 5%, 3—PCL/MMT 10%, 4—PCL/MMT 25%, 5—PCL/MMT 50%.

Inclusions of MMT in the granules prepared by solution-casting had dimensions of the same order. However, as more MMT was added in the PCL (10, 25 and 50 wt%), the number of pores and craters of 70–100 microns in size increased and the MMT particles tended to stick together and form agglomerates and tactoids up to 100–177 microns in size. According to the SEM images of the obtained granules, it can be noted that MMT was distributed in the polymer matrix in a random manner, resulting in the formation of a mixed composite structure, including the exfoliated composite. The microstructure of the granules was directly affected by the method for obtaining them. The PCL/MMT granules prepared by extrusion had a denser microstructure and fewer MMT agglomerates compared to the PCL/MMT granules prepared by solution-casting, exhibiting a loose microstructure. Moreover, for the granules prepared by solution-casting, noticeable anisotropy was observed in the SEM images that was expressed in differences in the microstructure on different sides of the cross-section of the corresponding granule (for example, inset 6 in Figure 2). Here, we assume that this feature of these samples was associated not so much with the inclusion of MMT, but with the preparation method, which implies gradual evaporation of the solvent resulting in irregularity in the spatial orientation of the polymer chains.

### 3.2. FTIR Spectroscopy

Figure 2 shows the IR spectra of the neat PCL and PCL/MMT samples prepared by solution-casting and melt extrusion. The spectra highlight the areas in which changes in the structure of the studied samples were observed depending on the preparation method and the amount of MMT.

**Figure 3:**
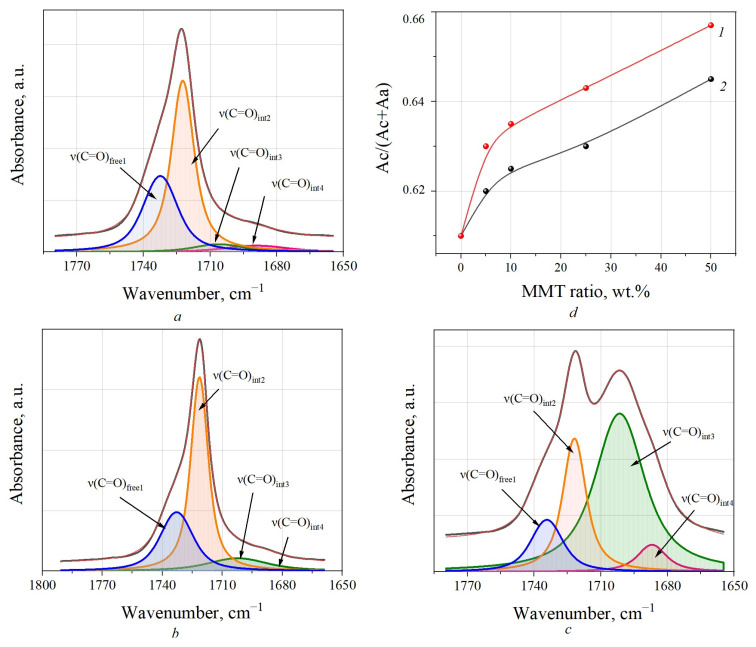
Deconvolution of FTIR spectra in the area of carbonyl groups C=O stretching vibrations for neat PCL (**a**), S_PCL/MMT 50% (**b**,1), E_PCL/MMT 50% (**c**,2), and dependence of the degree of crystallinity *X_c_* on the amount of MMT (**d**). The black line characterizes the experimentally obtained curve of the IR-Fourier spectrum, the red line reflects the result of deconvolution of the spectra.

For PCL, the absorption bands of the asymmetric stretch vibrations of the CH_2_ groups (2945 cm^−1^), the stretch symmetric vibrations of the CH_2_ groups (2867 cm^−1^), and the stretch vibrations of the C=O carbonyl groups (1723 cm^−1^) could be distinguished (Figure 3). For the PCL/MMT composites, vibrations of the -C-O-C- bond in the ester groups (1241, 1166 and 1047 cm^−1^), and bending vibrations of the -(CH_2_)x- fragments (733 cm^−1^) were observed. Moreover, as more MMT was added to the PCL, a prominent peak in the area of 570 cm^−1^ appeared, which can be attributed to the stretch vibrations of the Si-O bond present in the MMT.

IR spectroscopy is a highly sensitive tool for the detection of structural changes occurring during the crystallization of PCL. As can be seen in Figure 2, the peak from the C=O double bond in the ester group had an asymmetric shape with a pronounced shoulder. At the same time, for the PCL/MMT granules obtained by extrusion, as the amount of MMT increased, a change in the peak shape (for E_PCL/MMT 25%) and a clear division into two maxima (for E_PCL/MMT 50%) were visible. To obtain more details on the structural features of the ester group, the spectral contour in the 1800–1650 cm^−1^ area was decomposed into its elementary components (Figure 3). First, the number of elementary components and the positions of their maxima were determined by Fourier deconvolution. The obtained peak ν(C=O)free1 at 1732 cm^−1^ belonged to the carbonyl group free from intermolecular interactions in the amorphous phase of PCL. The ν(C=O)int2 peak at 1720–1722 cm^−1^ refers to the carbonyl group in the crystalline phase of PCL, and the appearance of ν(C=O)int3 and ν(C=O)int4 peaks at 1700 cm^−1^ and 1690 cm^−1^, respectively, may have been due to the formation of different crystalline domains of different size.

A graph showing the dependence of Ac/(Ac + Aa) on the amount of MMT is presented in Figure 3d. It is clearly seen that with increase in the amount of MMT, the crystallinity of the PCL/MMT granules increased in the case of both solution-casting and melt extrusion, but a more prominent increase was observed for the PCL/MMT granules prepared by solution-casting.

**Figure 4:**
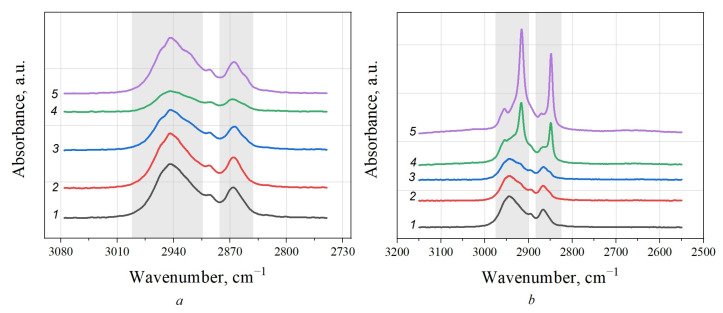
FTIR spectra in the stretch vibration area of CH_2_ groups (3080–2780 cm^−1^) for PCL granules prepared by solution-casting (**a**) and melt extrusion (**b**): 1—PCL, 2—PCL/MMT 5%, 3—PCL/MMT 10%, 4—PCL/MMT 25%, 5—PCL/MMT 50%.

The increase in the intensity of the ν(C=O)int3 and ν(C=O)int4 peaks may also have been due to the change in the electronegativity of the neighboring atoms due to the formation of C-H···O=C interactions. For example, Figure 4 shows the FT-IR spectra of the studied samples in the area of 3080–2780 cm^−1^ where methylene bands can be observed. As can be seen from the spectra of PCL/MMT granules prepared by extrusion, an increasing amount of introduced MMT also resulted in changes in the shape of the absorption bands of the -CH_2_ group, specifically, their shift to a low-frequency area, which confirms the formation of C-H···O=C interactions (Figure 4b) [33].

### 3.3. XRD Analysis

**Figure 5:**
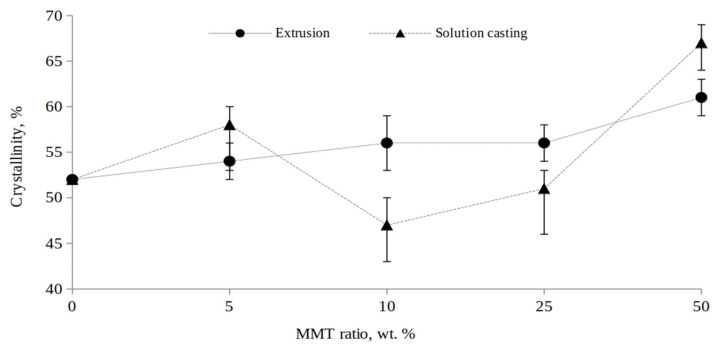
The degree of crystallinity of PCL/MMT granules obtained by different methods calculated by X-ray diffraction analysis.

**Figure 6:**
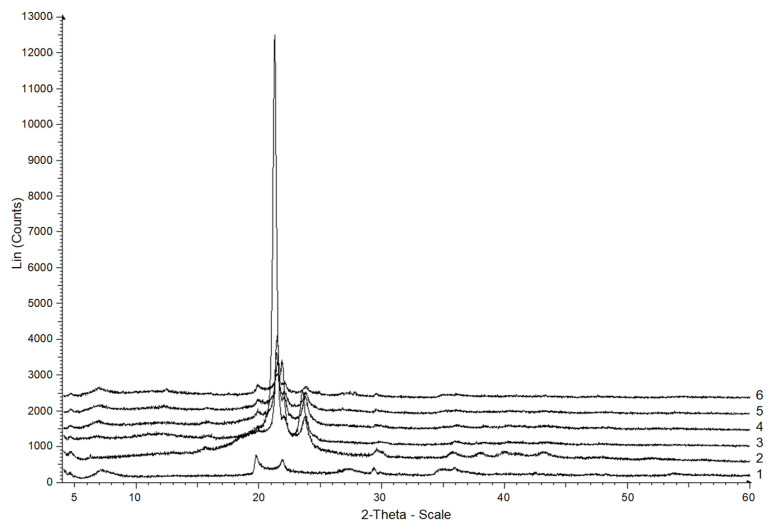
X-rays of MMT (1), PCL (2) and S_PCL/MMT granules prepared by solution-casting with different MMT content: 3—5%, 4—10%, 5—25%, 6—50%.

Highly crystalline MMT (measured *C_x_* = 55%) presented in the polymer matrix increased the crystallinity of the resulting composite PCL granules (Figure 5). Otherwise, the part of the polymer that entered into the boundary layer near the surface would not have crystallized, and crystallinity would have decreased. The crystallinity of the PCL/MMT granules prepared by extrusion ranged from 54 to 61% and was directly correlated with an increase in the MMT content in the polymer matrix (Figure 6). For the granules prepared by solution-casting, such a correlation was not observed when 10 and 25 wt% of MMT were included in the polymer matrix. The respective crystallinities of the granules reached 48 and 51%, while at the lowest and maximum content of MMT, they increased and reached 58 and 67%, which was confirmed by X-rays. The lack of correlation between the crystallinity and the MMT content in the solution-cast granules may have been due to the anisotropy of these samples, as mentioned above.

**Figure 7:**
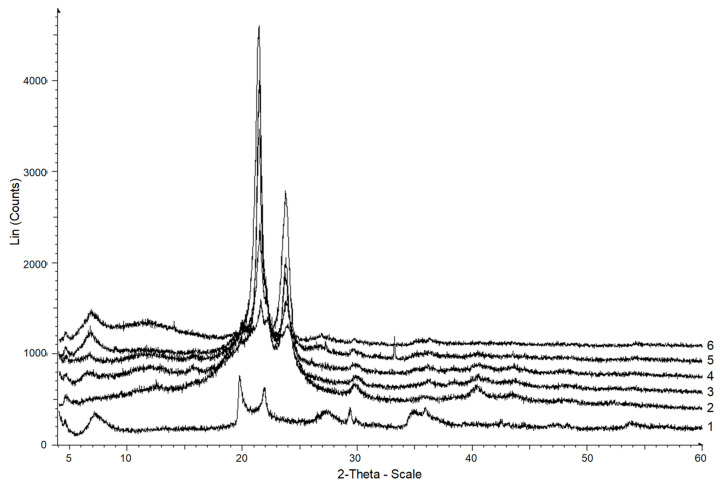
X-rays of MMT (1), PCL (2) and E_PCL/MMT granules prepared by extrusion with different MMT content: 3—5%, 4—10%, 5—25%, 6—50%.

The diffractograms of PCL (Figure 5, Figure 6, line 2) demonstrated an extensive amorphous halo with an angular width at 10–30°, and reflexes typical for PCL at 21.3°, 21.9° and 23.7°, which corresponded to the planes (110), (111) and (200) apparent on the X-rays. This confirms the polycrystalline structure of the polymer and corresponds to the existing data [12]. The MMT X-ray showed maxima with angular coordinates of 7.13°, complemented by an interlayer distance of 1.8 nm and two diffraction peaks at 19.71° and 21.87° (Figure 7), together corresponding to a basis reflection (001).

**Figure 8:**
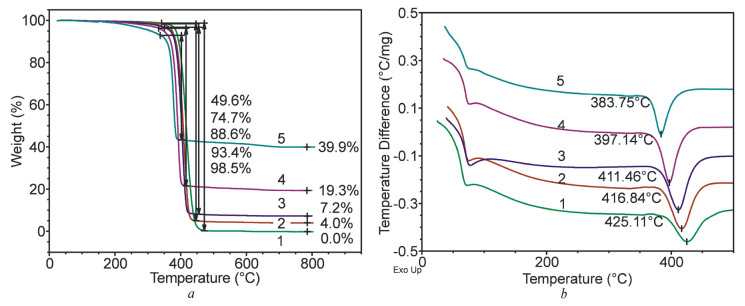
TGA (**a**) and DTA (**b**) curves of PCL (1) and E_PCL/MMT granules prepared by extrusion with different MMT content: 2—5%, 3—10%, 4—25%, 5—50%.

In the PCL/MMT granules prepared by solution-casting, the diffraction peaks shifted to 6.72°, 19.87°, and 21.98° (for S_PCL/MMT 5% and S_PCL/MMT 10%), and 22.03° (for S_PCL/MMT 25% and S_PCL/MMT 50%) (Figure 7). In the PCL/MMT granules prepared by extrusion, the diffraction peaks shifted to 6.42° (E_PCL/MMT 5%, E_PCL/MMT 10%), and 6.83° (E_PCL/MMT 25% and E_PCL/MMT 50%) (Figure 8), and there were no peaks at 19.87° and 21.98°.

An interval from 4° to 32.5° on Figure 7 shows that for E_PCL/MMT 5%, E_PCL/MMT 10%, E_PCL/MMT 25%, and E_PCL/MMT 50%, Fourier-smoothed curves (the considered maxima) are present with angles of 6.86°, 19.88°, and 22.02°. For the neat PCL granules (the Fourier curves were not smoothed) the maxima at 7° and 22° are absent, and at 20°, if there are any, they are hidden in the general rise of the curve.

The obtained X-rays confirm that the crystallization process of the PCL composite granules was affected not only by the content of MMT, but also by the method of obtaining the composite.

### 3.4. DSC and TGA

The obtained heat-flow data on neat PCL granules are presented in Table 1, along with the data on the crystallization temperature *T_c_* (°C) and the enthalpy of crystallization Δ*H_c_* (J/g), To estimate the crystallization enthalpy of PCL in the samples, its normalized values Δ*H_c_norm* (J/g) were calculated, taking into account the MMT content:

(6) $$\Delta H_{c}norm=\frac{\Delta H_{c}}{1-C_{MMT}}$$

where *C_MMT_* is the MMT content in a composite.

The inclusion of MMT in the PCL granules resulted in an increase in *T_c_* by 4.3–15.4 °C. A decrease in Δ*H_c_* by 4.2–23.1 J/g and a change in Δ*H_m_* from 64.4 to 25.3 J/g were also found. The crystallization parameters *T_c_* and Δ*H_c_* did not directly depend on the MMT content or the method of granule preparation. The decrease in Δ*H_c_* and the increase in *T_c_* of PCL/MMT granules indicate that the inclusion of the clay led to an increase in the degree of crystallinity of the PCL (Table 1). It is worth noting that the S_PCL/MMT 5% and E_PCL/MMT 50% samples stand out from the general trend. However, normalization of the obtained values to the MMT content (Δ*H_c_norm*) showed that the crystallinity of the polymer phase in the composite increased moderately as MMT was introduced in amounts of 5% and 10%, but decreased significantly when the MMT content increased to 50%. This may have been due to a decrease in the compatibility of the PCL and the MMT for a given amount of MMT.

The inclusion of MMT did not have a significant effect on the melting temperatures of the PCL composites, but significantly reduced the melting enthalpy (Table 1) by 1.0–1.8 times compared to PCL. The decrease in the melting enthalpy for the extruded granules was smooth and depended on the MMT content in the PCL composites, which was not typical for the granules prepared by solution-casting. Such difference in the melting enthalpy for the granules obtained by different methods indicates the influence of the method on the crystallization of PCL. A decrease in the melting enthalpy resulted from a denser packing of the macromolecules, the energy of intermolecular interaction of which was greater than before crystallization.

From the TGA and DTA data for granules prepared by melt extrusion (Figure 8a,b), it is clearly seen that all the samples showed a similar pattern of matrix polymer degradation. As the content of the filler increased, the degradation temperature of the polymer decreased from 425 °C to 383.7 °C. Quantitative data (such as the ash content and weight change during thermal degradation) showed a strong correlation with the corresponding PCL/MMT ratio, which indicates a high quality of the samples. The decrease in the degradation temperature with increase in the content of the filler was due to a catalytic effect of the surface of the filler.

**Figure 9:**
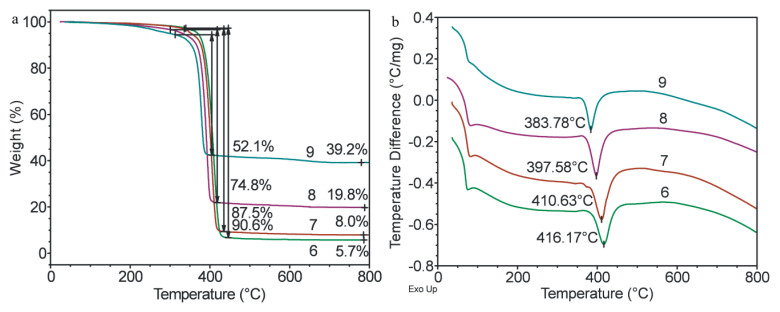
TGA (**a**) and DTA (**b**) curves of S_PCL/MMT granules prepared by solution-casting with different MMT content: 6—5%, 7—10%, 8—25%, 9—50%.

Study of the thermal properties of the granules prepared by solution-casting showed a similar pattern of thermal degradation. According to Figure 9, all the samples had a similar degradation pattern of the matrix polymer. As the content of the filler increased, the degradation temperature of the polymer decreased. Thus, an increase in the content of MMT resulted in a decrease in the processing temperature and the degradation temperature of the composite granules despite the method used for obtaining them.

### 3.5. Isothermal Crystallization Kinetics

In this study, the increase in the crystallinity of the composite granules occurred due both to the addition of highly crystalline MMT and to the appearance of spatial boundaries for the growth of the PCL crystals, resulting in the appearance of rather small and defective crystals. Small crystals are characterized by lower melting points, which may explain the decrease in the melting temperature with increase in the crystallinity.

To understand the differences in the crystallization rate of the PCL/MMT granules obtained by different methods, a process of isothermal crystallization was modeled for each sample. Table 2 shows the crystallinity, *n* and *t*_1/2_ values calculated using the Avrami equation from the DSC data [30,31,32].

In the study of bulk crystallization, we focused on three values: *n*, the Avrami exponent, which depends on the nucleation mechanism and the geometry of crystal growth; *t*_0_ which is the time of induction, defined as the time required to detect a significant increase in the conversion of the relatively early stage; and *t*_1/2_, or the time at which crystallization reaches 50% of completion. Taking into account the theory of nucleation and increase in the polymer crystallinity, the induction time can correlate with characteristics of the nucleation of the system, and *t*_1/2_ can be related to the kinetics of bulk crystallization.

If 1 < *n* ≤ 2, the growth is one-dimensional (rods); if 2 < *n* ≤ 3, the growth is two-dimensional (disks); if 3 < *n* ≤ 4, the growth is three-dimensional (spherulites). Integer values (2, 3 and 4) indicate homogeneous (uniform) growth, and fractional values indicate heterogeneous growth of the crystal structures [34].

The crystallinity, as measured by XRD (Figure 5) and DSC (Table 2) analyses, differed significantly (10–20%). However, in both cases, an increase in the MMT content led to an increase in the crystallinity of the composite granules. This suggests that for the literature data on the crystallinity of such composites, it is necessary to take into account not only the content and nature of the filler, but also the method of measurement to enable comparison with other studies.

At a cooling rate of 5 °C·min^−1^, the *n* values were different and random in nature. The *n* values reflecting heterogeneous one-dimensional growth were calculated for E_PCL/MMT 5% and E_PCL/MMT 50%, as well as for neat S_PCL, S_PCL/MMT 25% and S_PCL/MMT 50%. For the rest of the samples, the *n* values corresponded to heterogeneous two-dimensional growth. As for the crystallinity, there was a general trend in the observed increase, depending on both the MMT content and the cooling rate, with some exceptions (e.g., PCL/MMT 50% granules prepared by extrusion had a higher crystallinity at a higher cooling rate). For granules prepared by solution-casting, the addition of MMT slightly, and almost randomly, affected the crystallinity of the composites, while for the granules prepared by extrusion, the effect was clear and direct.

### 3.6. Biodegradation of the Composites in Soil

**Figure 10:**
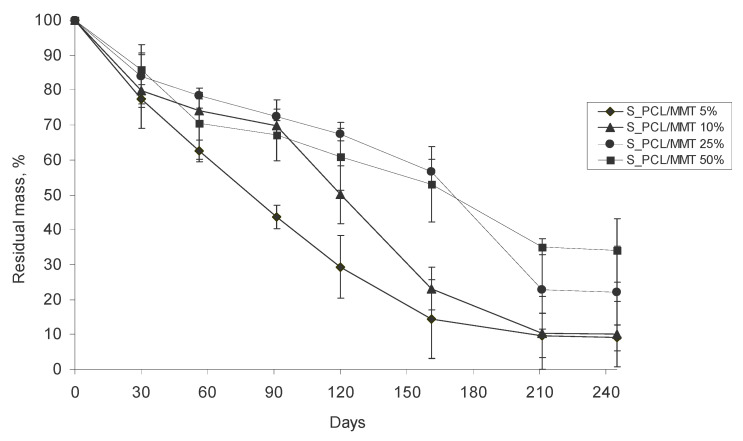
Mass loss of S_PCL/MMT granules prepared by solution-casting with different MMT content during their degradation in soil.

The biodegradation of polymer granules in soil occurs due to microbial activity, which depends on the ability of bacteria to produce enzymes which can facilitate the scission of complex polymer bonds. It was assumed that the inclusion of MMT in the PCL granules would correlate with mass loss of the granules and decrease the time for their degradation. However, according to the data on the mass loss of the S_PCL/MMT composite granules (Figure 10) obtained from the solution, we observed the opposite effect. Within 30 days, there were no significant differences in the residual masses between the granules with different MMT content. After 56 days, the S_PCL/MMT 5% granules degraded more rapidly, and on the 90th day, the residual mass of granules was 43.6%. In contrast, the equivalent values for S_PCL/MMT 10%, S_PCL/MMT 25% and S_PCL/MMT 50% were 69.8, 72.4 and 67.1%, respectively. On the 120th day of the experiment and up to the end, the S_PCL/MMT 5% granules appeared to demonstrate the greatest mass loss and degraded by 91.0%. The S_PCL/MMT 10% granules also degraded by 89%. The least rapid degradation from the 56th day and up to the end of the experiment was demonstrated for the S_PCL/MMT 50% and the S_PCL/MMT 25% granules. On the 245th day, the degrees of degradation for these granules were 66.0 and 73.4%, respectively. The corresponding residual masses were 34 and 26.6%.

**Figure 11:**
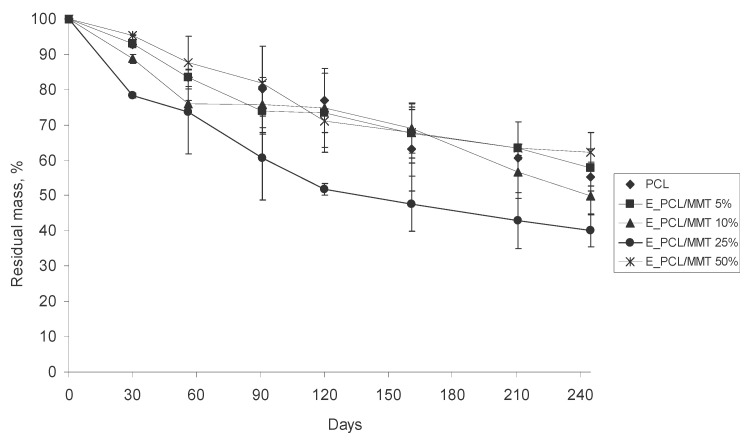
Mass loss of E_PCL/MMT granules prepared by extrusion with different MMT content during their degradation in soil.

The E_PCL/MMT granules prepared by extrusion generally degraded less rapidly during the whole experiment compared to the granules prepared by solution-casting (Figure 11). The greatest degradation during the whole experiment was observed for the E_PCL/MMT 25% granules. The remaining granules did not differ significantly in residual mass during the experiment. On the 245th day, the residual masses for the PCL, E_PCL/MMT 5%, E_PCL/MMT 10%, E_PCL/MMT 25%, and E_PCL/MMT 50% granules were 55.3, 57.9, 49.9, 40.1, and 62.3%, respectively. The greatest degradation of PCL/MMT 25% was probably due to the optimal concentration of clay and the activity of soil microbiota during the exposure of granules of this group in the soil. In contrast to the granules prepared by solution-casting, for the granules prepared by extrusion, no correlation was found between the PCL/MMT ratio and their biodegradation.

When comparing the effect of the preparation method on the mass loss of the composite granules, it was found that the use of the solution-casting method contributed to the formation of a more porous and loose structure in the granules in contrast to extrusion. The specific method used for the obtaining of granules also affects their crystallinity, which determines the spatial availability of the polymer for microbiota.

**Figure 12:**
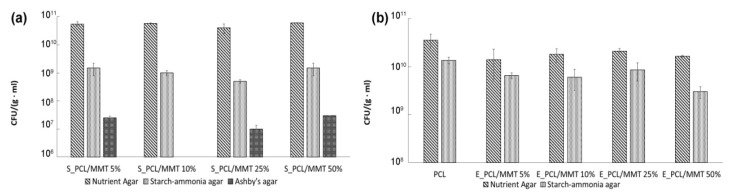
Microbial cell concentrations in suspensions of soil, where the PCL/MMT granules with different MMT content prepared by solution-casting (**a**) and by extrusion (**b**) were subjected to degradation.

The microbiological plating of suspensions of soil, where the granules were subjected to degradation, produced the following results: For the soil where the granules prepared by solution-casting were exposed, the growth in microorganisms was detected on nutrient agar, starch–ammonia agar and Ashby’s agar media (corresponding to ammonifiers, prototrophs that assimilate mineral nitrogen, nitrogen fixers and fungi) (Figure 12a). The number of ammonifiers did not differ significantly in all the soils where the S_PCL/MMT composite granules were exposed and amounted to approximately 5.4 × 10^10^ CFU/(g·mL). The number of prototrophs and nitrogen fixers in the soil after exposure of the S_PCL/MMT 25% granules was one order lower than in the other groups. The number of fungi was almost the same in all the groups, except for the soil where the S_PCL/MMT 10% granules were exposed. Regarding the Ashby’s medium, no growth was detected for all the S_PCL/MMT granules.

For the microbiological plating of suspensions of soil, where the granules prepared by solution-casting were exposed, the concentrations of ammonifiers and prototrophs considerably exceeded the equivalent concentrations for the soil, where the granules prepared by extrusion were exposed. However, after exposure of the E_PCL/MMT granules, nitrogen fixers and fungi were not observed (Figure 12b).

The maximal concentrations of ammonifiers and prototrophs were noted for the PCL granules without MMT and amounted to 3.5 × 10^10^ CFU/(g·mL) and 1.3 × 10^10^ CFU/(g·mL), respectively. The concentration of protorophs in this group much exceeded that for the other compositions. Among the rest of the E_PCL/MMT compositions, the concentrations of ammonifiers and prototrophs for the soil where E_PCL/MMT 25% granules were exposed were the highest, undoubtedly associated with the mass loss of the granules of this group. The absence of growth on the Sabouraud and Ashby’s agars probably has to do with the structure of the granules as they are quite dense, solid, and less porous than the granules prepared by solution-casting. Moreover, due to their porosity and the resulting availability of MMT for water, the S_PCL/MMT granules could absorb water, which is favorable for the growth of fungi.

## 4. Discussion

The PCL/MMT composite granules obtained in this study differed in the method for obtaining them. Granules prepared by solution-casting had a quite mixed structure and the MMT particles were randomly distributed in the matrix itself, as observed by SEM, in contrast to the intercalated structure of the granules prepared by extrusion. As stated previously, in the case of samples prepared by solution-casting (especially for the S_PCL/MMT 5% samples), a pronounced anisotropy of the structure was observed. As explained by Kida et al. (2023), this phenomenon is related to rapid thickening of the evaporating layer as well as the separation of the dense layers, shrinkage, and precipitation of the mixture components within the thickened layers when the evaporation rate is high. This can result in the loss of homogeneity of the sample structure [35].

When comparing the FTIR spectra of the PCL/MMT composite granules, only for the samples prepared by melt extrusion was a change in carbonyl and methylene bands observed. The increased intensity of the ν(C=O)int3 and ν(C=O)int4 peaks indicated the formation of more interactions between C-H and O=C groups for this type of granule compared with those prepared by solution-casting. This may have been due to differences in the orientation of the molecules in the melt inside the nozzle, which were subjected to additional shear strains than those in solution in the absence of external mechanical stress. The second factor is related to differences in the cooling rate of the melt and PCL solution.

Funaki et al. (2018) showed that during lamella formation in PCL, the formation of intermolecular interactions is primarily observed [33]. It can be assumed that more hydrogen bonds are formed during the extrusion of the PCL melt, which is characterized by higher entropy of the system. The formed hydrogen bonds lead to inhibition of the growth of the crystalline region after extrusion. Conversely, in the PCL solution, the formation of C-H···O=C interactions is hindered by the solvent molecules. Thus, the slow cooling rate and fewer hydrogen bonds promote the growth of crystals of a larger size.

Using XRD analysis and DSC, it is possible to determine the degree of clay dispersion. Usually, intense reflections in the range of 2θ = 3–10° indicate an intercalated ordered system with alternating polymer/silicate layers. However, when delamination occurs, that is, when individual silicate layers (1 nm thick) are uniformly dispersed in the matrix, there are no peaks present on the X-ray graphs due to loss of the structural identity of the clay [36].

The X-ray graphs of the PCL/MMT composite granules prepared by solution-casting are shown in Figure 6 and Figure 7. The characteristic peak (first peak) of MMT at a diffraction angle of 6.86°, which corresponds to a base distance of 4.29 nm, disappeared in composites with 5 and 10% MMT content. The absence of this peak was due to the delamination of the structure formed by too wide a space between the layers [37]. The layered structure was also confirmed by SEM. For composite granules prepared by extrusion, the MMT peak shifted towards a smaller angle, exhibiting an increase in the basal distance of 3 nm. An increase in the basal distance indicates that the silicate layers are well intercalated by the polymer chain [38]. The intensity of the basal diffraction peak increased with increase in the MMT content.

These phenomena directly affect the crystallinity of mixtures of relatively pure substances. According to the available data, the crystallinity of PCL composites is significantly affected by the nature and amount of the added filler. In a study by Chen and Evans [39], the crystallinity of PCL composites, with MMT, bentone 105 and bentone 111 as filler materials, obtained from the melt decreased from 40 to 37–38% at the maximum content of the filler (16 and 19%) and increased up to 45% at the minimum content (3–4%), depending on the used filler. According to Luduena et al., the crystallinity of PCL/MMT composites prepared by melt-processing decreased from 52 to 41% with the addition of MMT and increased to 53.7% as Cloisite 30B (modified MMT) was added [24]. In a study by Fukushima et al., the crystallinity of PCL composites obtained by hot-pressing with different clay fillers (CLOISITE 30B, NANOFIL 804, PANGEL S9) increased from 47 to 53% with 5% addition of the filler [16]. Clegg et al. demonstrated that the addition of Cloisite Na+ (CNA) and Cloisite 10A (C10A) clays into PCL films resulted in an increase in crystallinity from 52 to 55% [11]. In a study by Moussaif et al., the crystallinity of PCL/Cloisite30B and PCL/MCM-41 composites prepared by extrusion varied slightly from 45.7 to 46.8% [14]. In a study by Seyrek et al., the crystallinity of PCL/MMT and PCL/modified MMT composite films (0.5, 1 and 3% addition of MMT) prepared by solution-casting was studied by XRD analysis. It was found that as the MMT content in the composite films increased, the crystallinity of the composites decreased [40].

In our study, the crystallinity of the granules prepared by solution-casting directly depended on and correlated with the MMT content. The crystallinity of granules prepared by solution-casting with 10 and 25% MMT content decreased. Moreover, normalization of Δ*Hc* to the content of the polymer component in the granules (Table 1) showed that, with high MMT inclusion (50%), the crystallinity of PCL was significantly reduced. When modeling isothermal crystallization in melts (extruded granules), *t*_1/2_, as a parameter of the primary nucleation rate, was slightly lower than in granules prepared by solution-casting. However, when using different methods for obtaining them, it was observed that *t*_1/2_ was high only for 5 and 10% content of filler. With an increase in the MMT content, the *t*_1/2_ value dropped to 0.9, which was associated with a high amount of the clay. Luduena et al. demonstrated that the *t*_1/2_ value increased as the space between the layers became wider. The inclusion of C30B (modified MMT) in the PCL matrix led to an increase in *t*_1/2_, in contrast to the inclusion of unmodified MMT. The authors claimed that this was due to the compatibility between the matrix and the clay surface/modifier [24]. In a study by Krikorian and Pochan, a similar result was demonstrated for PLA/C30B nanocomposites, confirming the hypothesis that with improved clay/polymer compatibility, the dispersion of clay plates can interfere with chain folding for the local crystallization of PLA [41].

However, the prediction of possible options for the formation of polymer–silicate composites by compounding in solution and in melt is complicated by the influence of various factors on their structure and properties [42,43,44]. These include energy changes resulting from the penetration of the polymer into the clay galleries, changes in the spaces between the silicate layers, as well as parameters related to the intermolecular interaction between the silicate layers and interfacial polymer/clay/solvent interactions. Understanding these aspects in the obtaining of a composite can help to choose in advance the method for obtaining it and to predict the properties. The present study shows that understanding the choice of preparation method depends on the matrix itself and the nature of the filler. The use of solution-casting is less predictable compared to melt extrusion in terms of the properties of the resulting composite.

As a result of the biodegradation of the granules in soil, it was found that the method for obtaining them and the MMT content affected the mass losses of the granules. During exposure in soil, granules prepared by solution in some cases reached 90% of degradation, whereas granules prepared by extrusion degraded in less than 60%. The effect of the method of obtaining granules on their features, specifically, on their biodegradability, is probably related to the difference in the spatial arrangement of the polymer chains. In the extruded granules, they are arranged more densely and are closer to each other due to the pressure applied to the polymer mass during extrusion and the resulting availability to microorganisms.

Regarding the effect of the composition of the granules on their biodegradation, this was more evident for the granules prepared by solution-casting. For the granules prepared by extrusion, there was no direct correlation, except for the fact that the granules of both groups showed the minimal degradation for composites with 50% content of MMT. As noted above, the introduced MMT acted as a nucleating agent that assisted the appearance of native nuclei and, as a result, the increase in crystallinity. It is known that the increase in crystallinity, as well as the increase in crystal sizes and glass point of the polymer, negatively affect the ability of microbes to easily degrade polymer chains [45].

## 5. Conclusions

A comparative study of PCL/MMT composite granules prepared by solution-casting and melt extrusion with inclusion of montmorillonite was carried out. XRD analysis, FTIR spectroscopy and SEM showed that layers of the MMT exfoliate in the composites prepared by melt extrusion, and in the granules prepared by solution-casting, were randomly distributed in the polymer matrix. DSC analysis showed that the melting points of PCL/MMT composites were not strongly affected by the content of the filler and the method of obtaining them. However, the crystallization process was enhanced as MMT was added, which acted as an effective nucleation agent and was well-dispersed in the PCL matrix. According to the TGA and DTA data obtained, increase in the content of MMT resulted in a decrease in the processing temperature and the destruction temperature of the composite granules despite the method of obtaining the composites. The *t*_1/2_ values obtained by modeling the bulk crystallization mainly depended on the MMT content and were somewhat lower for granules prepared by extrusion than for granules prepared by solution-casting. The increase in crystallinity of the granules, depending on the method applied for obtaining them, also affected their degradation in soil. The mass losses of the granules prepared by solution-casting reached 90% in some cases, whereas for the extruded granules, this value was less than 60%. Possible applications of the studied PCL/MMT composite granules are as granules for the plastics industry or as delivery systems for various chemicals, including pesticides and fertilizers in agriculture due to their biodegradability and eco-friendliness. However, compared to composites prepared by melt extrusion, the more complex prediction of characteristics for composites prepared by solution-casting must be taken into account.

## Figures and Tables

**Table 1:** DSC data for PCL and PCL/MMT granules prepared by solution-casting and extrusion.

Sample	*T_c_*, °C	Δ*H_c_*, J/g	Δ*H_c_norm*, J/g	*T_m_*, °C	Δ*H_m_*, J/g
Solution-casting					
S_PCL	16.7	57.8	57.8	57.7	50.2
S_PCL/MMT 5%	27.0	42.5	44.7	56.8	38.2
S_PCL/MMT 10%	21.0	43.8	48.7	56.3	46.3
S_PCL/MMT 25%	23.0	39.4	52.5	55.8	25.3
S_PCL/MMT 50%	28.7	34.7	69.4	55.1	35.7
Extrusion					
E_PCL	14.2	55.0	55.0	57.0	64.4
E_PCL/MMT 5%	21.9	50.8	53.5	56.8	64.6
E_PCL/MMT 10%	27.0	43.7	48.6	56.7	62.7
E_PCL/MMT 25%	29.6	35.9	47.9	56.6	53.3
E_PCL/MMT 50%	27.7	45.3	90.6	54.6	36.4

**Table 2:** The results of applying Avrami’s equation to the obtained DSC data (heating rate = 5 °C·min^−1^) for the PCL/MMT granules.

Sample	Crystallinity, %	*n*	*t*_1/2_, min
Solution-casting			
S_PCL	39.69	1.8530	1.28
S_PCL/MMT 5%	41.84	2.3895	1.60
S_PCL/MMT 10%	41.20	2.2846	1.03
S_PCL/MMT 25%	45.23	1.6574	0.97
S_PCL/MMT 50%	48.66	1.7858	0.85
Extrusion			
E_PCL	40.67	2.5954	2.39
E_PCL/MMT 5%	45.84	1.7099	1.02
E_PCL/MMT 25%	45.36	2.1280	1.47
E_PCL/MMT 50%	46.55	1.6516	0.90

## Data Availability

The datasets generated during and/or analyzed during the current study are available from the corresponding author on reasonable request.
